# Risks of Colon Injuries in Extreme Lateral Approaches to the Lumbar Spine: An Anatomical Study

**DOI:** 10.7759/cureus.2122

**Published:** 2018-01-29

**Authors:** Emre Yilmaz, Joe Iwanaga, Marc Moisi, Ronen Blecher, Amir Abdul-Jabbar, Tamir Tawfik, Rod J Oskouian, R. Shane Tubbs

**Affiliations:** 1 Swedish Medical Center, Swedish Neuroscience Institute; 2 Seattle Science Foundation; 3 Neurosurgery, Wayne State University School of Medicine.; 4 Neurosurgery, Swedish Neuroscience Institute; 5 Neurosurgery, Seattle Science Foundation

**Keywords:** extreme lateral inter body fusion, xlif, colon injuries, spine surgery

## Abstract

Introduction

The extreme lateral interbody fusion technique (XLIF) is a modification of the retroperitoneal approach to the lumbar spine. This is a minimally invasive technique allowing direct access to the disc space without peritoneal or posterior paraspinal musculature damage. Nevertheless, the retroperitoneal part of the colon can be injured in this operative technique. To our knowledge, a study analyzing the anatomical considerations of the extreme lateral interbody fusion technique with regards to potential colon injuries has not been previously performed. Therefore, the aim of this study was to evaluate the potential risk of colon injuries during the extreme lateral approach to the lumbar spine.

Materials and Methods

The extreme lateral approach to the lumbar spine was performed on four fresh-frozen cadaveric sides. K-wires were placed into the intervertebral discs and positioned at L1/L2, L2/L3, L3/L4, and L4/L5 levels. Next, the distances from the wires to the most posterior aspect of the adjacent ascending or descending colon were measured.

Results

The mean distance from the intervertebral disc space to the ascending or descending colon was 23.2 mm at the L2/L3 level, 29.5 mm at the L3/L4 level, and 40.3 mm at the L4/L5 level. The L1/L2 level was above the colon on both sides.

Conclusion

Our study quantified the relationship of the retroperitoneal colon during an extreme lateral interbody fusion approach. Our results, as well as previously described cases of bowel perforations, suggest a greater risk for colon injuries at the L2/3 and L3/4 levels.​​​​​​​

## Introduction

Minimally invasive approaches to the anterior lumbar spine have evolved in order to improve treatment and reduce approach-related morbidity [1]. One such approach is the extreme lateral transpoas approach as first described by Ozgur et al. in 2006 [2]. The extreme lateral approach to the lumbar spine is a modification of the retroperitoneal approach to the lumbar spine. Intraoperative monitoring is used and the space between the 12th rib and the highest part of the iliac crest is entered. The extreme lateral interbody fusion technique is a minimally invasive technique allowing direct access to the disc space and without peritoneal or posterior paraspinal musculature involvement [3-7].

Despite its minimally invasive nature, the overall complication rate for the lateral approach based on the literature is about 18%. For example, weakness of the psoas major has been reported in 1-8% of patients following this procedure. Postoperative sensory nerve injury is reported to range from 5-49%. The most commonly reported injury is a nerve injury. Vascular and bowel injuries are rare complications associated with this approach [8]. However, Rodgers et al. found no vascular or intraoperative visceral injuries in 600 patients undergoing the lateral approach [9]. Nevertheless, the retroperitoneal parts (ascending and descending) of the colon can be injured with this approach [10, 11].

To our knowledge, a study analyzing the anatomical considerations of the extreme lateral approach to the lumbar spine and potential colon injuries has not been previously performed. Therefore, the aim of this cadaveric study was to evaluate the potential risk of colon injury during an extreme lateral approach to the lumbar spine. 

## Materials and methods

**Figure 1:**
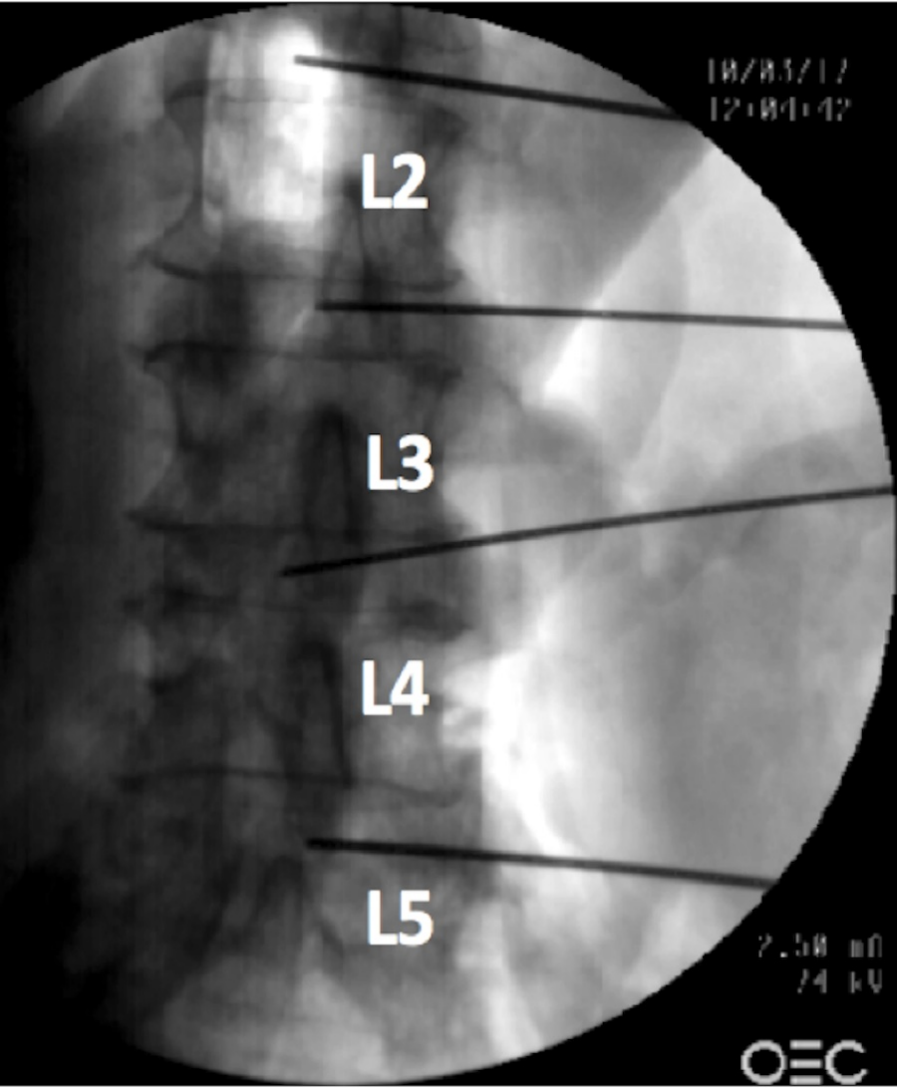
Labeled anteroposterior radiograph of the lumbar spine showing the placement of the wires which were used for the measurements.

**Figure 2:**
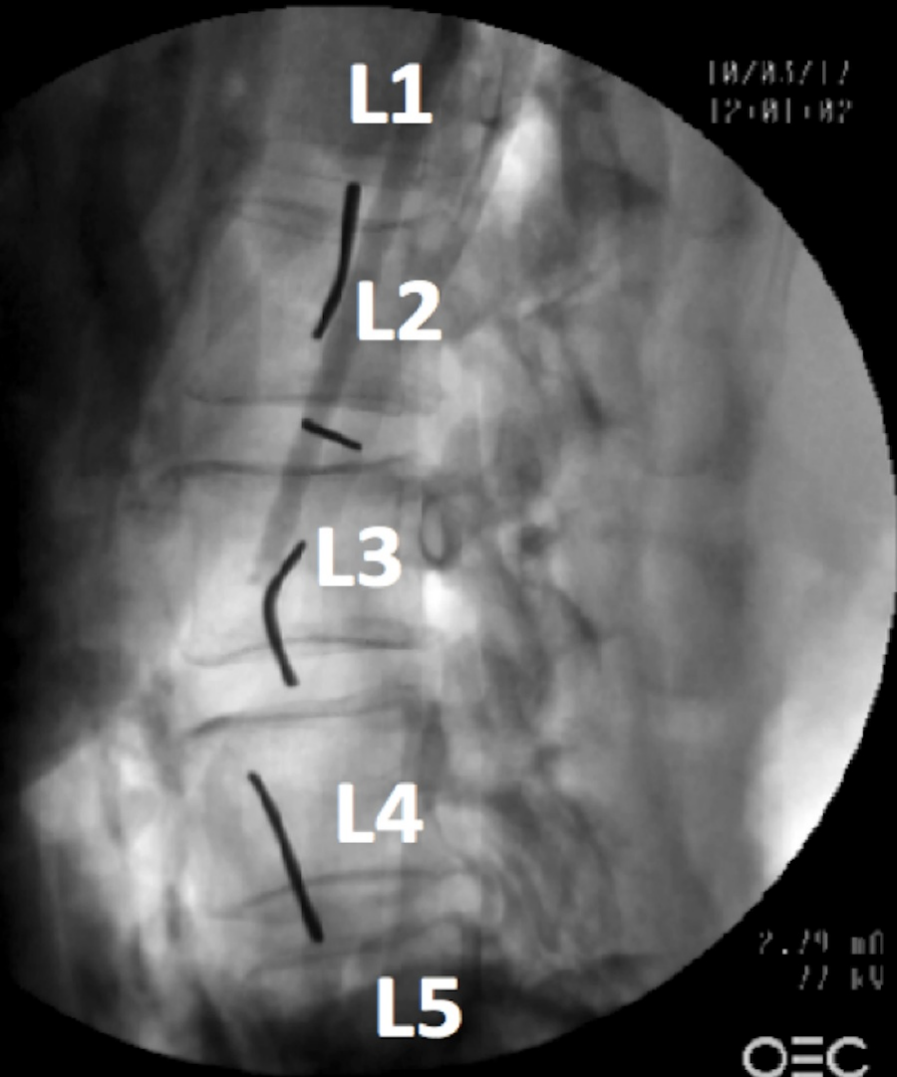
Labeled lateral radiograph of the lumbar spine showing the placement of the wires which were used for the measurements.

We performed an anatomical study on four sides from two fresh, frozen, and thawed adult cadavers (one male, 81 years at death; one female, 73 years at death) in a surgical training facility (Seattle Science Foundation, Seattle, Washington, USA). The dissections were initially performed in the prone position between the 12th rib and highest point of the iliac crest on each side. The latissimus dorsi muscle and the thoracolumbar fascia were dissected. The retroperitoneum was opened and the fat tissue was removed. Next, the cadavers were positioned in the direct lateral position (90°) and K-wires were placed into the intervertebral discs. The placement was confirmed using anteroposterior (Figure 1) and lateral fluoroscopy (Figure 2).

**Figure 3:**
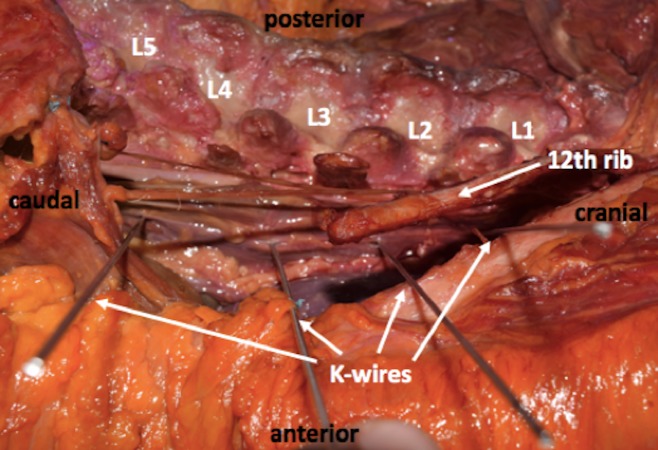
Lateral view showing the wire placement into the intervertebral disc spaces.

**Figure 4:**
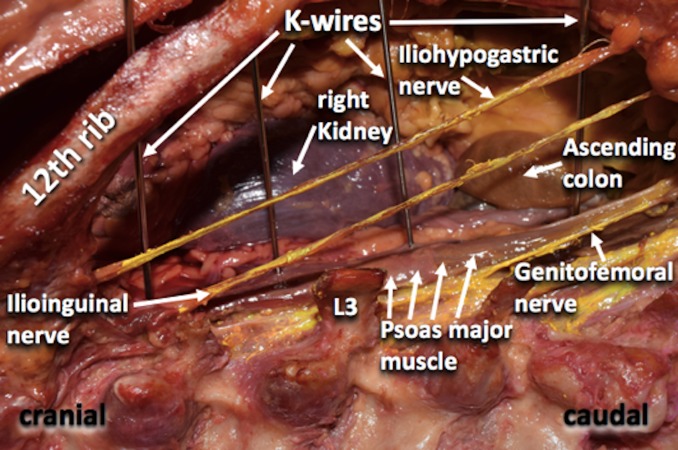
Posterior view of the retroperitoneal dissection.

All wires were placed by fellowship-trained spine surgeons. The wires were positioned at L1/L2, L2/L3, L3/L4, and L4/L5 levels. After this, the distances from the wires to the most posterior aspect of the adjacent ascending or descending colon were measured by two different surgeons and the average of the measurements taken (Figure 3, 4).

Measurements were made using microcalipers (Mitutoyo, Kanagawa, Japan) with a resolution of 0.01 mm and an accuracy of ±0.025mm.  

## Results

No scoliosis or other anatomical variants were observed in any of the specimens. No past surgical scars were noted in the areas dissected. No gross pathology or anatomical variations were seen in the areas dissected. The mean distance from the intervertebral disc space to the posterior edge of the ascending and descending colon was 23.2 mm (6.0-41.0mm) at the L2/L3 level, 29.5 mm (14.0-45.0mm) at the L3/L4 level and, 40.3 mm (20.0-60.0mm) at the L4/L5 level. The L1/L2 level was above the colon on both sides. No grossly visible differences were noted between the two specimens. These results are summarized in Table 1.

**Table 1:** Measurements from lumbar intervertebral disc spaces to the ascending and descending colon.

	Specimen 1 right side (mm)		Specimen 1 left side (mm)		Specimen 2 right side (mm)		Specimen 2 left side (mm)		
L1/L2	1	2	1	2	1	2	1	2	mean (mm)
Colon	X	X	X	X	X	X	X	X	X
L2/L3	1	2	1	2	1	2	1	2	mean (mm)
Colon	X	X	41	40	X	X	6	6	23.3
L3/L4	1	2	1	2	1	2	1	2	mean (mm)
Colon	X	X	14	14	X	X	45	45	29.5
L4/L5	1	2	1	2	1	2	1	2	mean (mm)
Colon	X	X	20	21	X	X	60	60	40.3

## Discussion

Minimally invasive approaches such as the extreme lateral lumbar interbody fusion have been shown to achieve similar or better outcomes in regards to pain and disability relief compared to direct approaches [8, 12-15]. Furthermore, studies suggest a lower complication rate with less blood loss, decreased risk of vascular or lumbar plexus injuries, decreased costs, and a shorter length of hospital stay [8, 9, 16-20].

Bowel injuries are rare, undesirable, and sometimes life-threatening complications that can occur after anterior lumbar spinal surgeries [9, 11, 21, 22].

In the present study, we analyzed the anatomical relations of the retroperitoneal parts of the colon to the intervertebral disc space of the lumbar spine in order to better understand the mechanisms leading to a bowel injury during the extreme lateral lumbar approach. We found that the ascending and descending colon are at risk, especially at the L2/L3 and L3/4 levels. The average distance from the disc space to the colon on left and right sides was 23.3 mm and 29.5 mm respectively. During a L4/L5 extreme lateral lumbar approach the distance was greater with an average distance of 40.3 mm from the disc space to the colon. Therefore, based on our cadaveric study, injury to the colon at this level would be less, compared to proximal lumbar levels during lateral approaches.

To our knowledge only two cases of bowel injuries after extreme lateral interbody fusion are described in the literature. Balsano et al. reported the case of a 70-year-old male who underwent an L3/4 and L4/5 extreme lateral transpsoas approach for interbody fusion. The patient suffered a perforation of the splenic flexure of the colon and required surgical intervention with a temporary colostomy for three months [10]. In their series, Tormenti et al. reported one bowel perforation out of eight scoliotic patients undergoing lateral transpsoas approaches. Specifically, a cecal perforation occurred, which necessitated an emergency exploratory laparotomy and bowel resection. As suggested by Tormenti et al., the rotatory component of scoliotic spines changes the topographical anatomy and could significantly increase the risk of damage to intra- and retroperitoneal structures [11]. In these cases, the preoperative imaging should be analyzed thoughtfully. The literature is lacking in studies analyzing topographical changes in scoliotic patients or analyzing anatomical variations related to the extreme lateral interbody fusion procedure.

To prevent injury of peritoneal and retroperitoneal components, complete access to the retroperitoneal space is necessary. The muscle fibers have to be carefully spread and dilators and retractors placed through the space of the lateral border of the psoas major muscle. Careful removal of the retractor and ensuring that there are no obvious injuries to the bowel are crucial.

## Conclusions

Our cadaveric study quantitated a close relationship of the retroperitoneal colon during extreme lateral interbody fusion procedures. Our results as well as the previous described cases of bowel perforations in the literature suggest a greater risk for injuries at the L2/3 and L3/4 levels during lateral approaches to the spine. Moreover, scoliotic spines might lead to a greater risk of colonic injuries.
